# South-West Orthopædic Club

**Published:** 1970-01

**Authors:** 


### South West Orthopædic Club

Nine members and two visitors of the South West Orthopædic Club spent a week in Orthopædic centres in Eire and Northern Ireland between the 19th and 23rd May, 1969.

### St. Mary's Orthopædic Hospital, Cork

**Mr. F. H. Moore** presented four cases of congenital absence of the fibula. This was really a dysplasia of the entire limb with short curved tibia and femur and dysplastic hip, foot abnormalities and a dimple over the tibia. Shortening could amount to five or six inches when fully grown. Syme's amputation, the most satisfactory treatment, should ideally be carried out at the age of one year and a pleasing prosthesis fitted with compensation for the shortening. The tibia then tended to straighten. **Dr. F. Dando** reviewed cases of tibial lengthening using Anderson's method. The average gain in length was two inches with bony union after four to five months. Freedom from pain was possibly due to the foot being allowed to go into equinus at first. Complications included pin track infection, skin sloughing and equinus deformities. **Mr. J. P. Golden** showed two cases of neuralgic amyotrophy of the shoulder girdle, a condition first described by Spillane. A man of twenty had paralysis of serratus anterior, deltoid and spinati. The serratus had remained paralysed but the other muscles had largely recovered. A man of sixty had complete paralysis of the spinati with no recovery. **Dr. N. Gallaghan's** paper on the neurological aspects of ischæmia of the spinal cord and cauda equina described the blood supply of the spinal cord which is in four segmental lengths divided by watershed areas. Ischæmic conditions can be due to arterial disease, cervical spondylosis, trauma or compressive lesions. The three clinical pictures of infarction of the spinal cord, intermittent claudication of the spinal cord, or cauda equina, sometimes associated with stenosis of the spinal canal were illustrated by **Mr. St. J. G. O'Connell** who described the treatment of a case of spinal stenosis by decompression laminectomy.

### St. Mary's Orthopædic Hospital, Cappagh, Dublin

**Mr. Boyd Dunlop** discussed the current problems of total prosthetic replacement of the hip, in particular the advantages and disadvantages of the Ring and McKee-Farrar operations and especially the difficulties of insertion of the guide drill in Ring's operation. The exact site of this guide could only be established with antero-posterior and closed and open oblique x-rays. After **Mr. J. Gallagher** had illustrated with patients some of the advantages of total hip replacement, there followed a general discussion on the whole question of total arthroplasty of the hip. **Mr. P. Macauley's** paper on non-union of tibial fractures described a technique for posterolateral exposure which he had used very successfully for dealing with non-union by autogenous cancellous grafting. Mr. Macauley then showed several patients who had undergone leg equalization procedures and the clinical part of the meeting was concluded by the demonstration of two cases of subluxation of the cervical spine, presented by **Mr. N. Mulvihill** and several rare bone dysplasias by **Mr. J. Gallagher.**

### Our Lady's Hospital For Sick Children, Crumlin, Dublin

**Mr. P. G. Brady** reported on no less than seven cases of diastematomyelia, all with an abnormal bar of bone in the lumbar region and associated with spina bifida, sometimes with hydrocephalus and usually a dimple or tuft of hair on the back. Some cases had hemivertebra or hip anomalies. The diagnosis was clearly made in all cases by myelogram. A film of an operation showed clearly the bone spike transfixing the spinal cord. This condition should be looked for in all cases of spina bifida. Removal of the bone spike prevented neurological deterioration and in some cases slight improvement ensued. **Mr. B. G. Regan** reported on his experience with spina bifida in Dublin where the incidence was 4.2 per thousand and he had received an average of 47 patients a year for the last six years. Despite early closure, the mortality was about 39% and all the survivors were permanent patients. Iliopsoas transplant, even if there was no evidence of hip subluxation, appeared to be very effective in correcting lumbar lordosis and flexion deformity of the hips. **Dr. M. Walsh** presented two children with pseudarthrosis of the tibia and neurofibromatosis. One had sound bony union after 4 operations; the other was still ununited after 3 bone grafts. He thought the fundamental fault was an intra-osseous neurofibroma interfering with osteoid. **Mr. Regan** then talked about the problems of instability of the hip in new born babies. There followed a discussion on the value of x-rays in infancy and the type and duration of splintage.

### Musgrave Park Hospital, Belfast

**Mr. R. I. Wilson** described the history of orthopædics in Northern Ireland, and the present organisation. A comprehensive service is given to the whole country, based on Belfast where there are nine consultant orthopædic surgeons. Londonderry has its own set-up with two consultants. There was a very useful link-up with Queen's University. **Mr. J. B. Pyper** had dealt with cartilage erosions of the femoral condyle and patella by removing the degenerate areas and using cartilage grafts from the good parts fixed in position with Smillie's pins. He had also treated a number of cases by multiple drilling, using the method of Pridie. Histology of the reformed cartilage after drilling showed that it was not normal hyaline cartilage but had a covering layer of fibrous tissue. **Dr. Jean Williamson,**

a research registrar, talked about the early diagnosis of congenital dislocation of the hip. She also discussed the ideal type of splint to be used and showed x-rays of a hip which remained dislocated in both Craig and Barlow splints. She therefore favoured the von Rosen type. In the prognosis of hip instability the angle of abduction at which the hip reduces is an important factor. A child with a poor acetabulum which only reduces in wide abduction may well need more careful and prolonged treatment. **Mr. B. T. Crymble** discussed some problems arising in the treatment of established congenital hip dislocation. The head of the femur might not sink into the acetabulum properly or might not come down properly with traction. There might be good early reduction but later subluxation. Operative treatment was described.

**Mr. A. I. Macafee** gave a retrospective analysis of femoral neck fractures. Of 496 patients, the majority were women between 70 and 85. 74 per cent were operated on and the operations were equally distributed between Smith-Petersen pinning, nail plating and prosthetic replacement; 15 per cent died in hospital. Orthopædic complications occurred in 28% of Smith-Petersen pinnings, 6% of nail platings and 12% of replacements. The length of stay in hospital was highest with conservative treatment and lowest with replacements. The cost was approximately £300 per patient in 1967. **Mr. A. R. Gurd** read a preliminary communication on his research into the diagnosis of fat embolism. **Mr. O. H. Osterberg** gave an account of four generations of a family with eleven cases of a bone dysplasia. The average age of onset was twenty-three. The condition affected different parts of the skeleton and a definite diagnosis had not been made. Some of them resembled polyostotic fibrous dysplasia and some Paget's disease but they were not really typical of either. Histological reports, when available, suggested fibrous dysplasia. Two of them had developed malignant change and there had been four amputations. He was still studying this family and there were now 73 children under 16, so that a number of further cases were expected.

This tour was arranged by Mr. N. Mulvihill, the secretary of the Irish Orthopaedic Association.

